# TAp73-Mediated the Activation of C-Jun N-Terminal Kinase Enhances Cellular Chemosensitivity to Cisplatin in Ovarian Cancer Cells

**DOI:** 10.1371/journal.pone.0042985

**Published:** 2012-08-10

**Authors:** Pingde Zhang, Stephanie Si Liu, Hextan Yuen Sheung Ngan

**Affiliations:** Department of Obstetrics and Gynecology, LKS Faculty of Medicine, The University of Hong Kong, Hong Kong SAR, People's Republic of China; Vanderbilt University Medical Center, United States of America

## Abstract

P73, one member of the tumor suppressor p53 family, shares highly structural and functional similarity to p53. Like p53, the transcriptionally active TAp73 can mediate cellular response to chemotherapeutic agents in human cancer cells by up-regulating the expressions of its pro-apoptotic target genes such as PUMA, Bax, NOXA. Here, we demonstrated a novel molecular mechanism for TAp73-mediated apoptosis in response to cisplatin in ovarian cancer cells, and that was irrespective of p53 status. We found that TAp73 acted as an activator of the c-Jun N-terminal kinase (JNK) signaling pathway by up-regulating the expression of its target growth arrest and DNA-damage-inducible protein GADD45 alpha (GADD45α) and subsequently activating mitogen-activated protein kinase kinase-4 (MKK4). Inhibition of JNK activity by a specific inhibitor or small interfering RNA (siRNA) significantly abrogated TAp73-mediated apoptosis induced by cisplatin. Furthermore, inhibition of GADD45α by siRNA inactivated MKK4/JNK activities and also blocked TAp73-mediated apoptosis induction by cisplatin. Our study has demonstrated that TAp73 activated the JNK apoptotic signaling pathway in response to cisplatin in ovarian cancer cells.

## Introduction

P73, a novel member of the tumor suppressor p53 family, is similar to p53 both structurally and functionally [1], [2]. The p73 gene encodes more than 20 protein isoforms due to the usage of different promoters and alternatively post-transcriptional splicing. The transcriptionally active TAp73 isoforms, containing full N-terminal transactivation domain, can bind specifically to p53 responsive elements and transactivates some of the p53 target genes, and subsequently induce cell cycle arrest and apoptosis, while the DNp73 isoforms, with truncated N-terminal transactivation domain, acts as a dominant-negative inhibitor of both TAp73 and p53 [1], [3], [4]. Interestingly, TAp73 is also a mediator of cellular sensitivity to chemotherapeutic agents in human cancer cells [1], [4]–[7]. Many pro-apoptotic genes, such as PUMA, Bax and NOXA, act as activators of the mitochondrial apoptotic pathway, and have p73 responsive elements in their promoter and can be up-regulated by p73 to induce apoptosis in response to chemotherapeutic drugs. In addition, p73-mediated up-regulation of the death receptor CD95, a mediator of the extrinsic apoptotic pathway, also contributes to p73-mediated apoptosis in cancer cells under stress stimuli [8]. Yet, unlike p53, the molecular mechanisms implicating in p73-mediated cellular apoptosis are still not clearly understood. Understanding the precise underlying molecular mechanisms will be useful in targeting p73 as a good candidate gene for cancer therapy.

The JNK belongs to a superfamily of mitogen-activated protein (MAP) kinases. The JNK protein kinases contain Jnk1, Jnk2 and Jnk3. Jnk1 and Jnk2 are ubiquitously detectable. The Jnk3 is mainly restricted to brain, heart and testis [9]. The JNK signaling pathway responses to various stress stimuli, through the transduction of the upstream MAPKKK including MEKKs, and subsequently activation of JNK by phosphorylated at Thr and Tyr sites by the JNK direct upstream kinases MKK4/MKK7. Activation of JNK phosphorylates and activates the downstream transcription factor c-Jun and other transcription factors [9], [10]. The JNK signaling pathway acts as a key positive modulator of cell apoptotic response to stress stimuli [9]–[11]. In addition, the JNK signaling pathway contributes critically to cisplatin-dependent apoptosis in cancer cells [12]–[15].

In this study, we aimed to study the effect of TAp73 (TAp73α) on cellular response to cisplatin in ovarian cancer cells and the underlying molecular mechanisms. We were interested in whether TAp73 would have any regulatory role in other apoptotic pathways, such as the JNK signaling pathway, upon cisplatin treatment.

## Results

### TAp73α enhances cellular sensitivity to cisplatin in ovarian cancer cells

**Figure 1:**
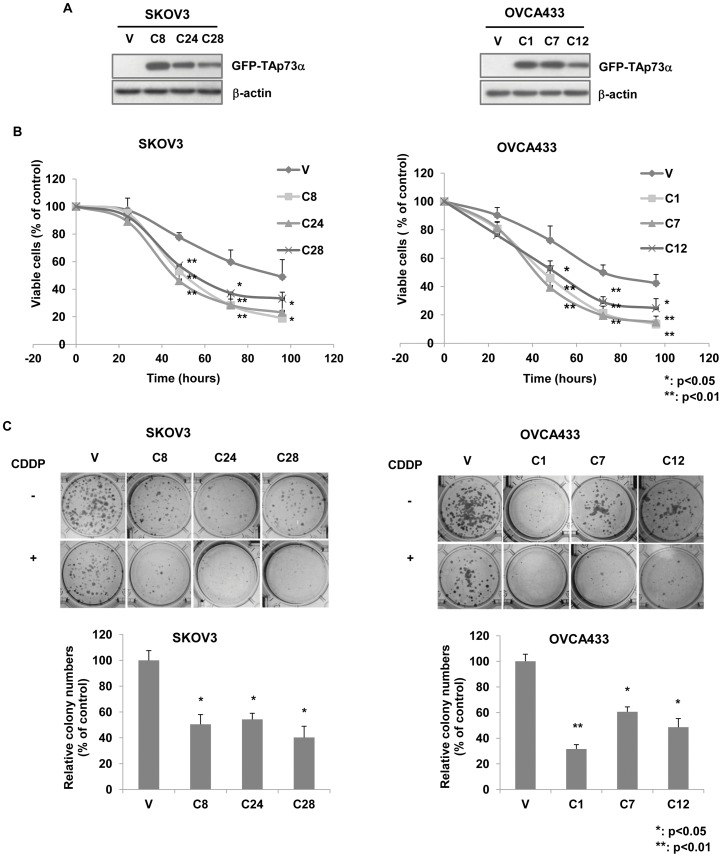
Overexpression of TAp73α enhanced cellular sensitivity to cisplatin. (A) The GFP-TAp73α overexpressing stable clones in SKOV3 (C8, C24 and C28) and OVCA433 (C1, C7 and C12) cells were verified by western blot analysis. (B and C) Both XTT viability assay and clonogenic assay showed significantly reduced cell proliferation in TAp73α-overexpressed cells of SKOV3 and OVCA433 compared to the empty vector controls (V) in response to cisplatin treatment. The percentage of cells/colonies surviving in cisplatin relative to cells/colonies in drug-free medium control was measured. Data was shown as mean ± SD from three independent experiments (*: p<0.05; **: p<0.01).

**Figure 2:**
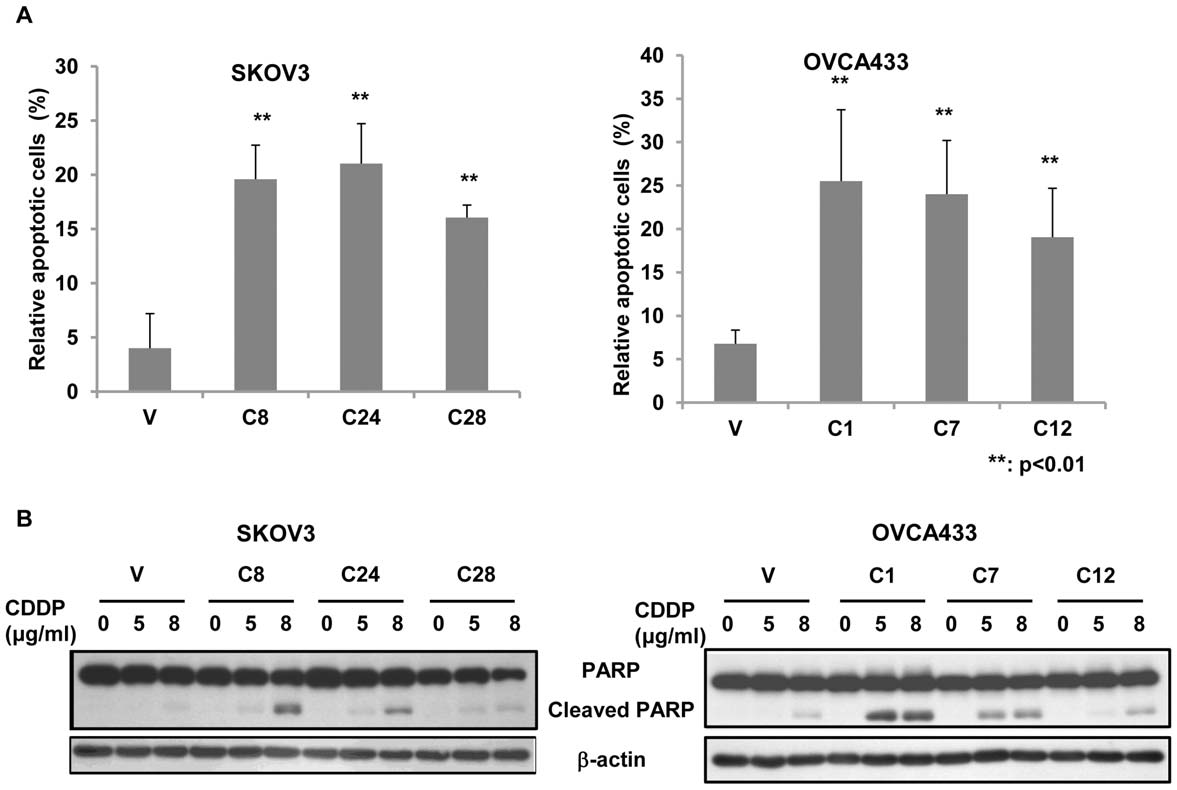
Overexpression of TAp73α promoted cell apoptosis in response to cisplatin. (A) TAp73α-overexpressed cells (SKOV3 C8, C24 and C28 and OVCA433 C1, C7 and C12) and the empty vector controls (V) of SKOV3 and OVCA433 were treated with 4 μg/ml cisplatin for 48 h. Apoptotic cells were assessed by TUNEL assay. More than 500 cells were counted for each group, the results presented were the relative of the apoptotic cells to total cells and at least three independent experiments were performed (**: p<0.01). (B) TAp73α-overexpressed cells and empty vector controls were treated with different doses (indicated) of cisplatin for 24 h, and the cleavage of PARP was detected by western blot analysis.

To investigate the role of TAp73 in ovarian cancer cells in response to cisplatin, human cisplatin-resistant ovarian cancer cell lines SKOV3 (null-p53) and OVCA433 (wild-type p53) were stably transfected with the plasmid pEGFP-TAp73α (Figure 1A). The effect of TAp73α on cellular response to cisplatin was assessed by both XTT cell viability assay and clonogenic assay. As shown in Figure 1B and 1C, TAp73α significantly increased cellular sensitivity to cisplatin in both null-p53 SKOV3 and wild-type p53 OVCA433 cells, when compared to the vector controls. Such effect was observed in both short-term (by XTT assay) and long-term (by clonogenic assay) culture assays. Furthermore, cell apoptosis induced by cisplatin was also increased by over-expression of TAp73α, as evidenced by TUNEL assay and cleaved PARP expression analysis (Figure 2A and 2B). These results indicated that TAp73 promoted cellular sensitivity to cisplatin via the induction of cell apoptosis, and such TAp73 function was p53-independent, as the effects were similar in both wild-type p53 and null-p53 cells.

### TAp73α mediates the activation of JNK signaling pathway

**Figure 3:**
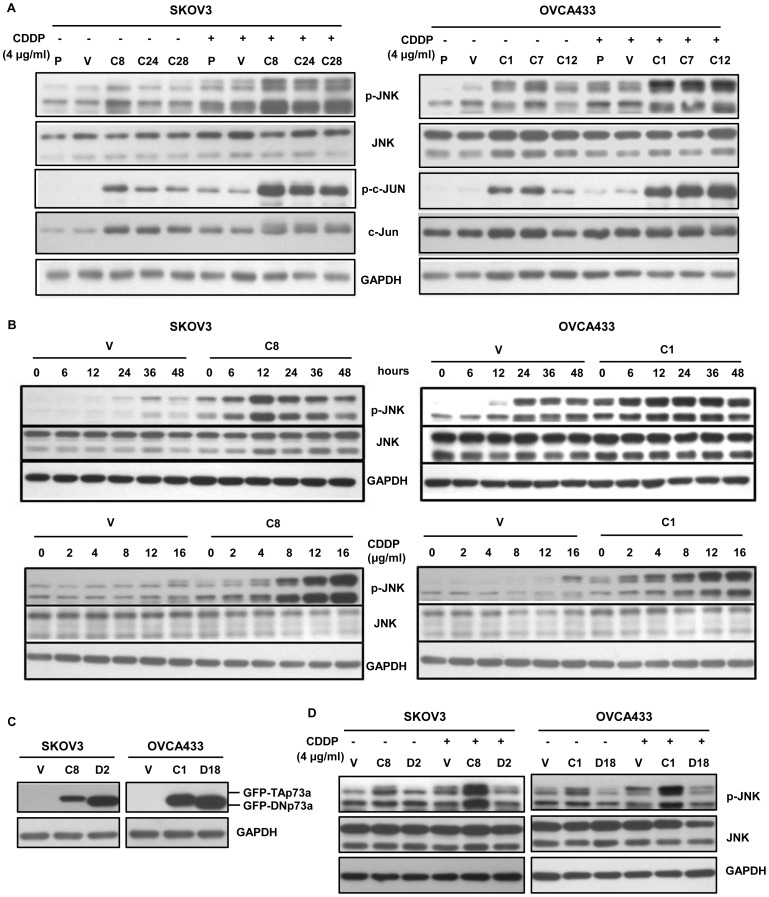
TAp73α activated the JNK pathway. (A) Increase of p-JNK and p-c-Jun was detected in TAp73α–overexpressed cells (SKOV3 C8, C24 and C28 and OVCA433 C1, C7 and C12) and further enhanced upon cisplatin treatment (4 μg/ml for 24 h), when compared to the controls (P: parental cells; V: empty vector control). (B) TAp73α-overexpressed cells (SKOV3 C8 and OVCA433 C1) were exposed to 4 μg/ml cisplatin for different periods of time, or to different doses of cisplatin for 12 h. The p-JNK level was measured by western blot analysis. (C) DNp73α (GFP-DNp73α) was over-expressed in SKOV3 (D2) and OVCA433 (D18) cells. (D) No JNK activation was observed in DNp73α-overexpressed cells (SKOV3 D2 and OVCA433 D18).

Previous reports have shown that activation of JNK contributes critically to cisplatin-induced cell apoptosis [12]–[15]. We thus hypothesized that TAp73α-mediated cell apoptosis in response to cisplatin might act through the activation of JNK signaling pathway. The effect of TAp73α on the activation of JNK signals was firstly analyzed by measuring the phosphorylation level of JNK (p-JNK) and its substrate c-Jun (p-c-Jun) in TAp73α-overexpressed cells. As shown in Figure 3A, both p-JNK and p-c-Jun were obviously elevated in TAp73α-overexpressed cells, when compared to the control cells. The increase of p-JNK and p-c-Jun were further augmented in these cells in response to cisplatin, only a slight increase of p-JNK and p-c-Jun was observed in the control cells. The time and dose-dependent experiments demonstrated that cisplatin-induced JNK activation occurred at as early as 6 h, and up to 48 h after the cells treated with 4 μg/ml cisplatin, and the effective cisplatin dosage for JNK activation was at as low as 2 μg/ml for 12 h treatment in TAp73α-overexpressed cells (SKOV3 C8; OVCA433 C1; Figure 3B). In addition, the activation of JNK was dependent on the transactivational activity of TAp73α as the p-JNK level was not changed in the cells stably over-expressing DNp73α (Figure 3C and 3D), a truncated isoform of p73 without transactivation activity. To verify whether the effect of TAp73α overexpression in promoting the cellular sensitivity was specific to cisplatin, we also performed the similar experiments with the treatment of Taxol, which is also a first-line anticancer drug used for ovarian cancer treatment. We found that Taxol was not able to activate JNK pathway in TAp73 overexpressed ovarian cancer cells, although TAp73α could enhanced the cell sensitivity in response to Taxol (Figure S1).

### Inhibition of JNK activity attenuates TAp73α-mediated apoptosis in response to cisplatin

**Figure 4:**
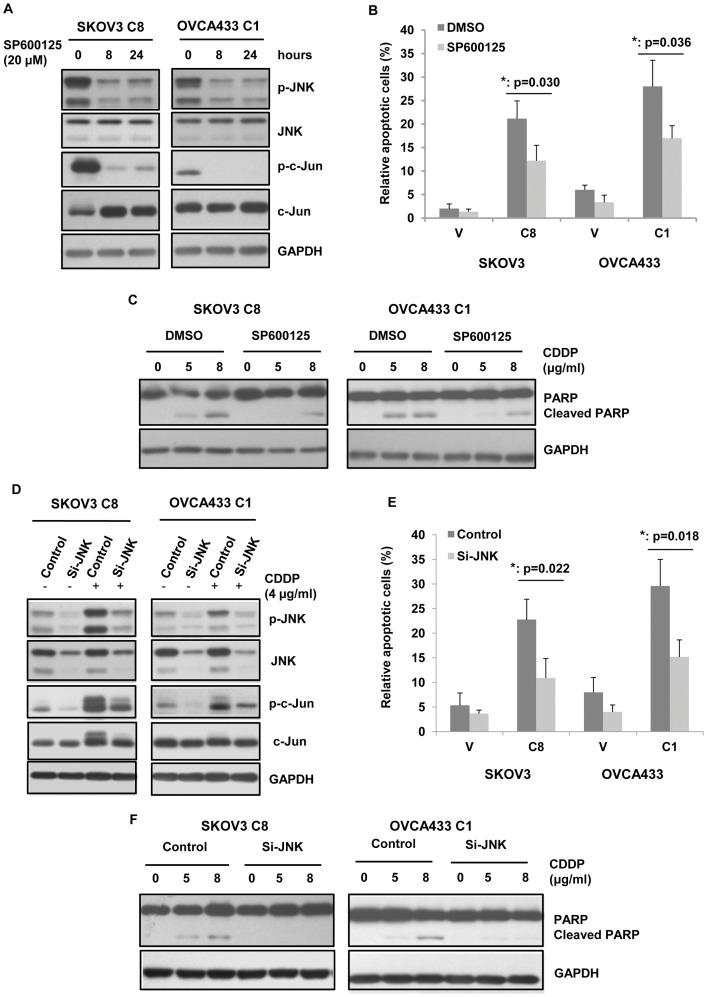
Activation of JNK was involved in TAp73α-mediated apoptosis induced by cisplatin. (A) TAp73α-overexpressed cells (SKOV3 C8 and OVCA433 C1) were treated with 20 μM SP600125 for different periods of time (indicated). The phosphorylation levels of JNK and c-Jun were measured. (B and C) TAp73α-overexpressed cells (SKOV3 C8 and OVCA433 C1) and the empty vector controls (V) were treated with 20 μM SP600125 or DMSO and then with cisplatin. The cell apoptosis were assessed by TUNEL assay (error bars indicated mean ± SD from three independent experiments; *: p<0.05) and the cleavage of PARP analysis. Inhibition of JNK attenuated TAp73α-mediated apoptosis in response to cisplatin. (D) TAp73α-overexpressed cells (SKOV3 C8 and OVCA433 C1) were treated with JNK siRNAs (Si-JNK) or the scrambled control siRNA (Control). The activations of JNK and c-Jun were absent upon cisplatin treatment, and associated with markedly reduced cell apoptosis (E and F).

To further explore whether TAp73α-mediated the activation of JNK contributed to apoptosis induction in response to cisplatin, a specific inhibitor of JNK kinase activity, SP600125, was used. After treatment of 20 μM SP600125 for 8 hours, the p-JNK level was reduced up to 60% in the TAp73α-overexpressed cells (SKOV3 C8 and OVCA433 C1, Figure 4A). In addition, inhibition of JNK activation by SP600125 significantly reduced cell apoptosis induced by cisplatin in TAp73α-overexpressed cells, as evidenced by TUNEL assay and cleaved PARP expression analysis (Figure 4B and 4C).

Activation of the JNK pathway by TAp73α implicated in cisplatin-induced apoptosis was also demonstrated by the observation that silence of JNK1 and -2 in TAp73α-overexpressed cells significantly blocked cisplatin-induced cell death. As shown in Figure 4D, treatment of siRNAs against JNK1 and -2 in cancer cells (SKOV3 C8; OVCA433 C1) obviously down-regulated JNK1/2 expression compared to the scrambled siRNA controls, and the treatment subsequently suppressed the phosphorylation levels of JNK and its substrate c-Jun. As evidenced by TUNEL assay and cleaved PARP expression analysis, the cisplatin-induced apoptosis in TAp73α-overexpressed cells (SKOV3 C8; OVCA433 C1) was significantly attenuated by JNK silencing treatment (Figure 4E and 4F). These results further confirmed that the activation of JNK pathway by TAp73α contributed to TAp73α-mediated apoptosis in ovarian cancer cells in response to cisplatin.

### TAp73α-mediated up-regulation of GADD45α is responsible for the activation of JNK signaling pathway

**Figure 5:**
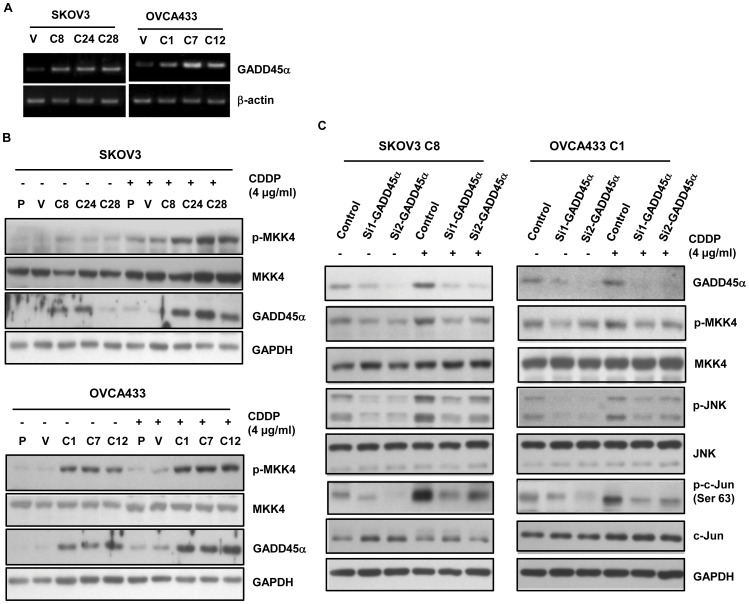
TAp73α activated the JNK pathway through up-regulating GADD45α and the subsequent activation of MKK4. (A) Increase of GADD45α mRNA expression in TAp73α-overexpressed cells (SKOV3 C8, C24 and C28 and OVCA433 C1, C7 and C12). (B) Increase of GADD45α protein expression and the MKK4 phosphorylation level in TAp73α-overexpressed cells (SKOV3 C8, C24 and C28 and OVCA433 C1, C7 and C12). (C) GADD45α was knocked down in TAp73α-overexpressed cells (SKOV3 C8 and OVCA433 C1) by siRNAs (Si1-GADD45α and Si2-GADD45α) treatment. The activation of MKK4, JNK and c-Jun were diminished, even under the cisplatin treatment.

GADD45α has been identified as a binding partner and activator of the JNK upstream kinase MEKK4/MTK1, and its binding to MEKK4/MTK1 can activate the downstream gene MKK4 and JNK [17], [18]. On the other hand, GADD45α is a well-defined target gene of p73 [19], [20]. Thus, we hypothesized that GADD45α might play a role in the activation of JNK signaling pathway mediated by TAp73. The effect of TAp73 on GADD45α expression was first assessed. As shown in Figure 5A and 5B, the expression of GADD45α was up-regulated in both mRNA and protein levels in TAp73α-overexpressed cells. In response to cisplatin, TAp73α-mediated up-regulation of GADD45α protein was further increased (Figure 5B). As GADD45α can directly interact with MEKK4/MTK1 to activate its substrate MKK4 kinase [17], [18], we then measured the phosphorylation level of MKK4 in TAp73α-overexpressed cells. As shown in Figure 5B, increase of phosphorylated MKK4 was observed in TAp73α-overexpressed cells and further enhanced in response to cisplatin treatment. These results suggested that TAp73α mediated the activation of JNK signaling pathway possibly through up-regulation of its target gene GADD45α and subsequent activation of MKK4, an upstream component of JNK signaling pathway.

To further verify these findings, a pair of siRNAs against GADD45α (Si1-GADD45α and Si2-GADD45α) was used to transiently knockdown the GADD45α expression. The phosphorylation levels of MKK4, JNK and c-Jun were accordingly reduced upon the down-regulation of GADD45α, even in response to cisplatin treatment (Figure 5C). Therefore, we confirmed that TAp73α-mediated up-regulation of GADD45α was responsible for the activation of the JNK signaling pathway in ovarian cancer cells.

### GADD45α is responsible for TAp73α-mediated apoptosis

**Figure 6:**
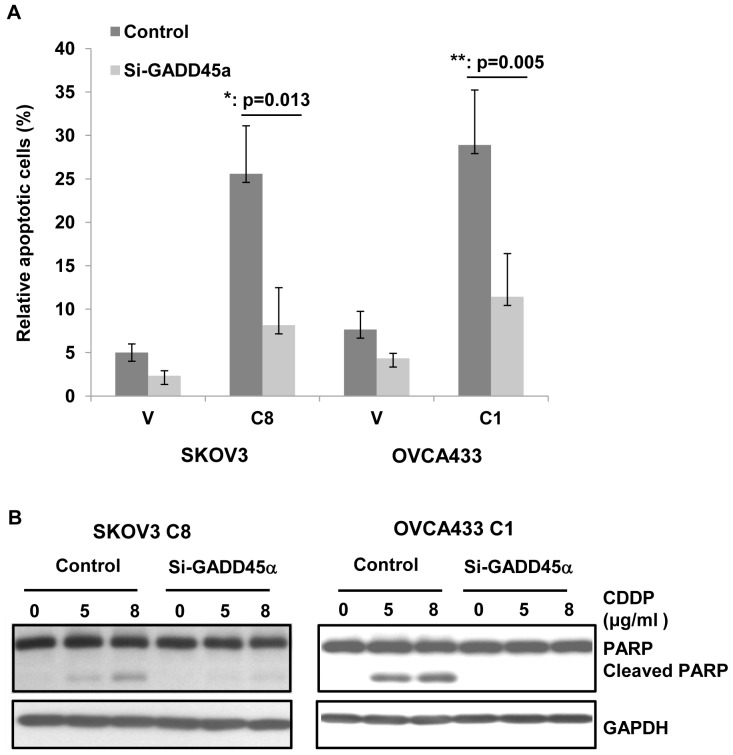
GADD45α contributed to TAp73-mediated apoptosis in response to cisplatin. Cell apoptosis was diminished in TAp73α-overexpressed cells (SKOV3 C8 and OVCA433 C1) in response to cisplatin after GADD45α siRNAs treatment. The cell apoptosis was measured by (A) TUNEL assay (error bars indicate mean ± SD from three independent experiments; *: p<0.05; **: p<0.01) and (B) the cleavage of PARP assay.

To determine whether silence of GADD45α has an effect on cisplatin-induced apoptosis in TAp73α-overexpressed cells, cells were treated with GADD45α siRNAs and cell apoptosis was measured. As shown in Figure 6, cisplatin-induced apoptosis was dramatically decreased in GADD45α-silenced TAp73α-overexpressed cells (SKOV3 C8; OVCA433 C1). These findings suggested that TAp73α-mediated the up-regulation of GADD45α was responsible for the activation of the JNK signaling pathway and cell apoptosis in ovarian cancer cells.

## Discussion

In this study, we demonstrated that, for the first time, to our knowledge, TAp73 partially mediated cellular apoptosis in response to cisplatin through the activation of JNK signaling pathway in ovarian cancer cells. We found that, in response to cisplatin, TAp73α activated the JNK signaling pathway via the up-regulation of its downstream gene GADD45α, and the response was TAp73-dependent and p53-independent.

Established evidence has shown that the transcriptionally active TAp73 enhanced cellular sensitivity to chemotherapeutic drug cisplatin in human cancer cells [5]–[7], [21]. In addition, a recent report has demonstrated that TAp73 expression in ovarian cancers was much higher in responsive cancers compared with unresponsive cancers [22]. Our study confirmed that TAp73 did enhance cellular sensitivity to cisplatin in ovarian cancer cells. Although TAp73 is functionally similar to p53, functional p53 expression has been shown to be necessary for TAp73-mediated cell apoptosis under DNA damage stimulation [23]. On the other hand, some studies suggested that p73-mediated chemosensitivity is independent of the p53 expression in some cancer cells [5], [24]. In our present study, TAp73α-mediated apoptosis was observed in p53-null SKOV3 cells, indicating that TAp73-mediated apoptosis was p53-independent, at least in our cell model, further emphasizing the independent role of p73 among its family members in DNA damage response.

The JNK cell death pathway functions as an important regulator of cell apoptosis in response to various stress stimuli and actually plays a central role in apoptotic pathways, including extrinsic (death receptors) [11] and intrinsic (mitochondrial) pathways [11], [25]. Previous studies have shown that cisplatin-mediated activation of the JNK signaling pathway in cancer cells contributed critically to cisplatin-dependent apoptosis [12]–[15]. Up-regulation of Fas L expression resulted from activation of JNK and its substrate c-Jun played a key role in cisplatin-induced apoptosis in ovarian cancer cells [15]. Our results showed that JNK and its substrate c-Jun were activated in TAp73α over-expressed cells, and further augmented in response to cisplatin treatment. Suppression of JNK activation by JNK inhibitor or JNK siRNAs in these cells abrogated TAp73α-mediated apoptosis. These results suggested that up-regulation of JNK activity contributed to TAp73-mediated apoptosis induction in ovarian cancer cells in response to cisplatin. This was the first time to show that TAp73 functioned as an upstream activator of the JNK pathway to activate JNK signals to induce cell apoptosis.

To further explore the underlying mechanisms by which TAp73 up-regulated the JNK phosphorylation level, a p73 target gene GADD45α was aware. GADD45α is a well-defined downstream gene of TAp73 and p53 [19], [20] and it can be induced by DNA damage agents, and plays an important role in the induction of apoptosis [26]. Interestingly, previous studies have shown that the activation of JNK apoptotic pathway by GADD45α was closely implicated in GADD45α-mediated apoptosis induction [27], [28]. GADD45α directly interacted with MEKK4/MTK1 to activate the substrate MKK4 kinase, and consequently up-regulate the JNK activation, an event that is involved in apoptosis induction [17], [18]. Our study has demonstrated that the expressions of both GADD45α and active MKK4 were up-regulated in TAp73α-overexpressed cells, and this effect was further augmented after cisplatin exposure. On the other hand, when GADD45α was silenced, the activations of MKK4, JNK and c-Jun were abolished, even under the treatment of cisplatin, and the TAp73α-mediated cisplatin-induced apoptosis was also significantly blocked. These results indicated that TAp73 was able to activate the JNK apoptotic pathway by up-regulating GADD45α expression in ovarian cancer cells in response to cisplatin.

**Figure 7:**
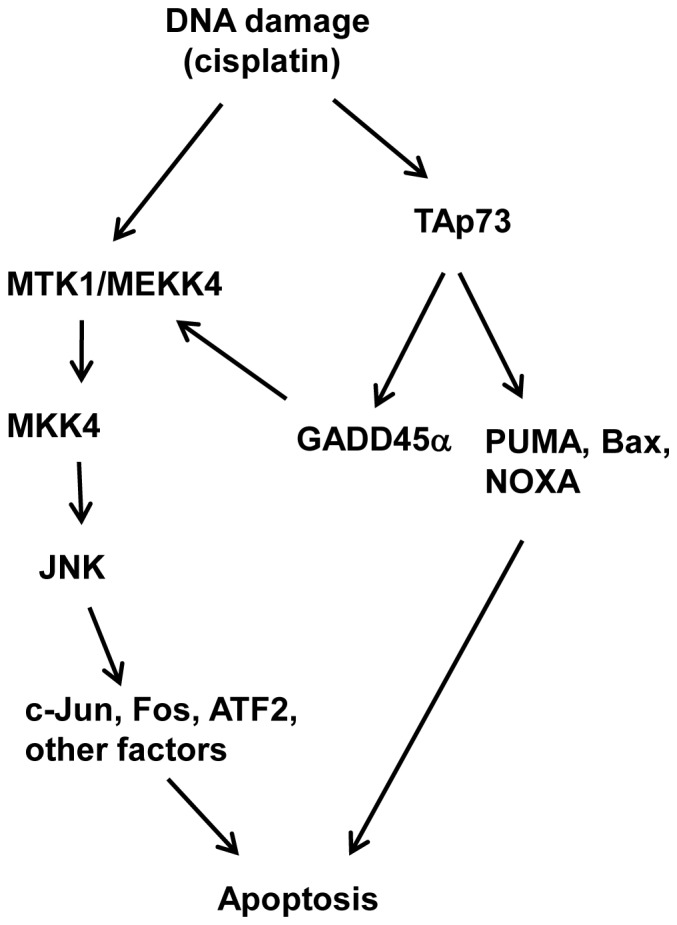
A proposed model for the regulatory role of TAp73 in DNA damage response. DNA damage agent, cisplatin induces TAp73 accumulation and the subsequent up-regulation of its pro-apoptotic target genes to induce cell apoptosis. Simultaneously, up-regulation of TAp73 target gene, GADD45α activates the JNK apoptotic pathway via its interaction with MEKK4/MTK1 to induce apoptosis.

Interestingly, previous report has proposed a cross talk between the JNK pathway and p73, and suggested that JNK was an important regulator in p73-mediated apoptosis in response to cisplatin [13]. They showed that p73 was a substrate of JNK kinase. JNK phosphorylated p73 at certain sites to enhance p73 transcription activity and p73-mediated apoptosis in cancer cells in the presence of cisplatin. In the present study, we demonstrated that over-expression of TAp73 activated the JNK activity in ovarian cancer cells, in response to cisplatin. TAp73 acted as an upstream activator of the JNK apoptotic pathway via the up-regulation of GADD45α, and the subsequent activation of MKK4 (Figure 7). Thus, our results provided a new angle to the cross talk between p73 and the JNK pathway in cell apoptosis response.

Collectively, our results clearly support a novel pathway of TAp73-mediated cellular sensitivity to cisplatin in ovarian cancer cells. We demonstrated that TAp73α induced the apoptotic response through the activation of the GADD45α-MKK4-JNK cell death cascade and provided a novel scenario for the cross talk between p73 and the JNK apoptotic pathway under stresses. This TAp73-dependent and p53-independent cellular response would play an important role in DNA damage response in ovarian cancer cells, as p53 function was defective in most of the cancer cells. A better understanding of the underlying mechanisms in TAp73-mediated apoptotic response is valuable in targeting p73 as a therapeutic candidate gene.

## Materials and Methods

### Cell culture and drug treatment

Human ovarian cancer cell lines SKOV3 (null p53) and OVCA433 (wild-type p53) were the gift from Prof. Tsao, Department of Anatomy, the University of Hong Kong, where SKOV3 was obtained from ATCC, Manassas, VA [29], and OVCA433 was established and described previously [30]. They were maintained in Minimum Essential Medium (MEM) (Invitrogen Corporation, Grand Island, YN), with 10% Fetal Bovine Serum (Invitrogen), and incubated in a 37°C humidified incubator containing 5% CO_2_.

Cis-Diamineplatinum(II) dichloride (Cisplatin, CDDP) (Sigma-Aldrich Corp., St. Louis, MO), and the JNK-specific inhibitor SP600215 (Calbiochem, San Diego, CA) were dissolved in milli-Q water and dimethyl sulfoxide (DMSO) (Sigma), respectively, and then stored at −20°C. These solutions were further diluted in culture medium before cell treatment. DMSO was diluted in medium alone as the control.

### Plasmids, siRNA and transfection

The pEGFP-TAp73α and pEGFP-DNp73α plasmids were constructed by PCR amplification of the full length coding region of TAp73α and DNp73α on cDNAs of ovarian cancer cells. And the PCR products were digested and then cloned in frame into the pEGFP expression vector (Clontech laboratories, Mountain View, CA). For stable clones, the pEGFP-TAp73α and pEGFP-DNp73α transfectants were selected by G418 (Invitrogen) for 14 days and then the single colonies were picked up and verified by western blot analysis. The siRNAs against JNK and GADD45α and the scrambled siRNA (negative control) (Applied Biosystems by Life Technologies, Foster City, CA) were transfected to cells at 20 nM final concentration. Lipofectamine 2000 (Invitrogen) was used for cell transfection according to the manufacturer's instructions.

### RT-PCR

Total RNA of cells was extracted using Trizol reagent (Invitrogen), and cDNA was synthesized with High Capacity RNA-to-cDNA Master Mix kit (Applied Biosystems). The specific primers (GGAGGAAGTGCTCAGCAAAG and TCCCGGCAAAAACA AATAAG) were used to amplify human GADD45α cDNA.

### Western blot analysis

Cells were harvested with 0.05% trypsin/EDTA (Invitrogen) and proteins were extracted using conventional RIPA lysis buffer [16]. The antibodies against GFP, JNK and GADD45α were purchased from Santa Cruz Biotechnology Inc. (Santa Cruz, CA). The antibodies against MKK4, PARP and c-Jun and the active forms of JNK, c-Jun (ser63) and MKK4 were obtained from Cell Signaling Technologies (Beverly, MA).

### Cell viability analysis

Cell viability was assessed by XTT (2,3-bis [2-methoxy-4-nitro-5-sulfophenyl]-2H -tetrazolium-5-carboxanilide inner salt) kit II (Roche Diagnostics GmbH, Mannheim, Germany) according to the manufacturer's protocol. Briefly, cells were seeded in triplicate in 96-well plate and treated with cisplatin (4 μg/ml) at the following day. The cell viability was measured after one day of treatment for four consecutive days.

### Clonogenic assay

The colony-forming ability of cells was measured by clonogenic assay. Cells were plated in triplicate in medium containing cisplatin (0.15 μg/ml) or drug-free medium at a concentration of 500 cells per well in 6-well plate. After 48 h incubation, all of these cells were allowed to grow in drug-free medium for 10–12 days. Surviving colonies were fixed in 75% ethanol and then stained in 1% giemsa (Merck, Damstadt, Germany). Colonies consisting of more than 50 cells were counted.

### Apoptosis assay

Apoptotic cells were examined by TUNEL assay (In Situ Cell Death Detection Kit, Roche) according to the manufacturer's instructions. Briefly, Cells were seeded on coverslips one day before 4 μg/ml cisplatin treatment for 48 h. After fixation and permeabilisation, cells were stained with TUNEL reaction mixture, and counterstained with DAPI (4′,6-diamidino-2-phenylindole) (Sigma).

### Statistical analysis

Data was expressed as mean ± SD of three independent experiments. SPSS 16.0 software (SPSS) was used for data analysis. Student's t-test was used to assess the difference between groups. A P value <0.05 was considered statistically significant.

## Supporting Information

Figure S1 **TAp73α enhanced cellular sensitivity to Taxol, but not via the JNK pathway.** (A) XTT viability assay showed TAp73α-overexpressed cells of SKOV3 and OVCA433 were more sensitive to Taxol treatment, compared to the empty vector controls (V). (B) After cisplatin or Taxol treatment for 24 h, the phosphorylation level of JNK was not increased in SKOV3 and OVCA433 cells treated with Taxol, even with TAp73α over-expression cells (C: control; D: cisplatin: T50 and T500: 50 ng/ml and 500 ng/ml Taxol).
(TIF)
