# Supplementary figures and images for: TAp73-Mediated the Activation of C-Jun N-Terminal Kinase Enhances Cellular Chemosensitivity to Cisplatin in Ovarian Cancer Cells
Source: PLoS One. 2012 Aug 10;7(8):e42985. doi: 10.1371/journal.pone.0042985 (PMC3416758; doi:10.1371/journal.pone.0042985)

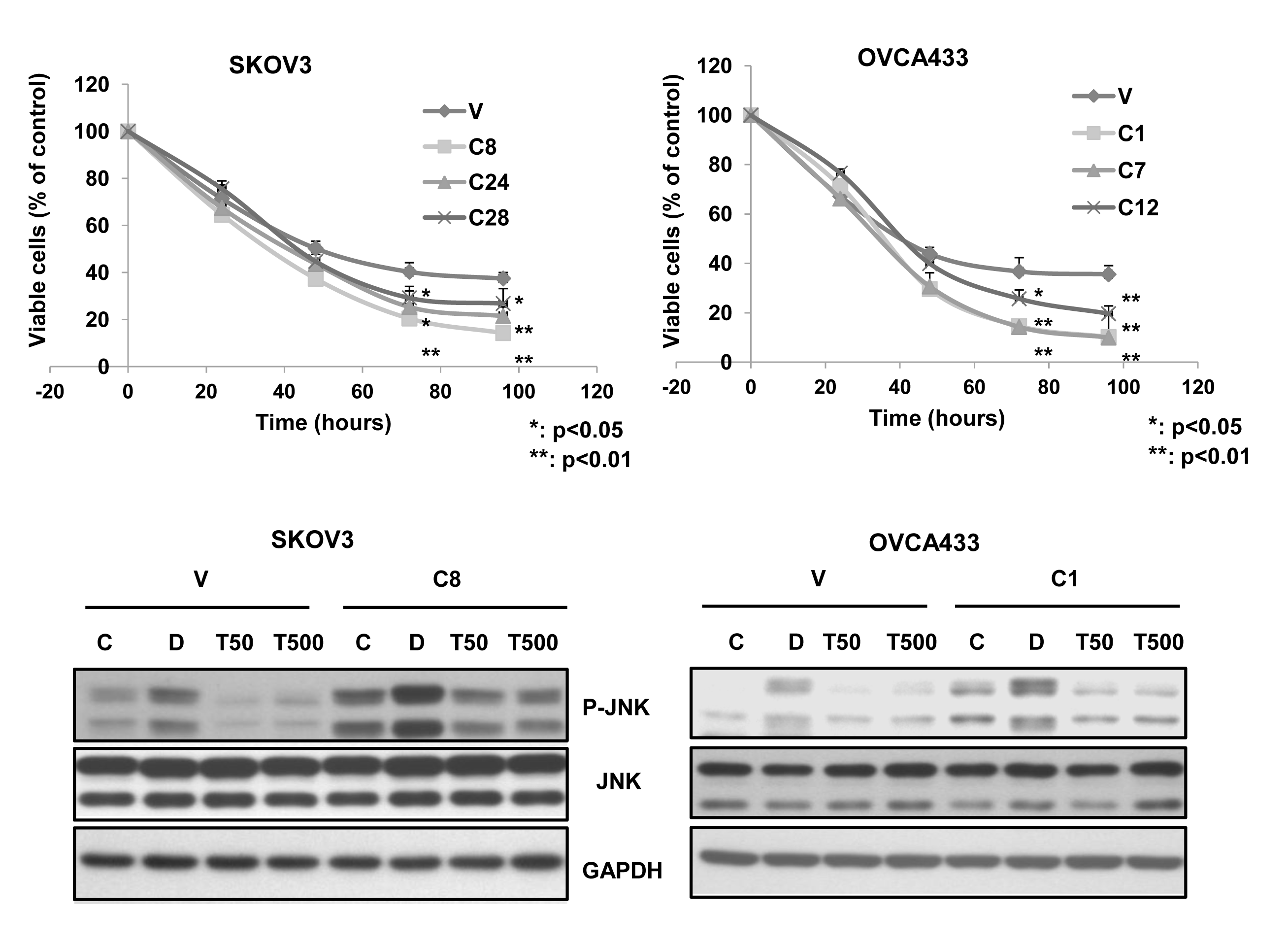

Supplement: Figure S1 — TAp73α enhanced cellular sensitivity to Taxol, but not via the JNK pathway. (A) XTT viability assay showed TAp73α-overexpressed cells of SKOV3 and OVCA433 were more sensitive to Taxol treatment, compared to the empty vector controls (V). (B) After cisplatin or Taxol treatment for 24 h, the phosphorylation level of JNK was not increased in SKOV3 and OVCA433 cells treated with Taxol, even with TAp73α over-expression cells (C: control; D: cisplatin: T50 and T500: 50 ng/ml and 500 ng/ml Taxol). (TIF) [file pone.0042985.s001.tif]
